# Combined exercise programs as protective factor against depression later in life: A systematic review

**DOI:** 10.1192/j.eurpsy.2021.1129

**Published:** 2021-08-13

**Authors:** K. Argyropoulos, E. Ntantouti, A. Argyropoulou, D. Avramidis, E. Jelastopulu

**Affiliations:** 1 Medical School, School of Medicine, University of Patras, Patras, Greece; 2 Postgraduate Program “aging And Chronic Diseases Management”, Joint Degree, School Of Medicine, University Of Thessaly & Hellenic Open University, Hellenic Open University, Patras, Greece; 3 General Practice, Health Center of Andravida, Patras, Greece; 4 Department Of Public Health, Medical School, University of Patras, Patras, Greece

**Keywords:** Depression, combined exercise, exercise modality, Elderly

## Abstract

**Introduction:**

Exercise has been repeatedly reported as an effective means of preventing and treating mood disorders. Therefore, there is a significant research interest for the way exercise is connected with depression and the effectiveness of different exercise parameters as intensity, duration and modality. There is significant research evidence supporting the hypothesis that exercise can alleviate the symptoms of clinical depression. Nevertheless, there has not enough evidence to compare the effectiveness of deferent types of exercise as complementary therapy in depression.

**Objectives:**

The purpose of the present study was to review the available research concerning the effect of exercise modality in depression and attempt to code and analyze the programs used in elderly (>65).

**Methods:**

A systematic review was contacted of randomized control trials published in electronic journals. The electronic data bases PubMed, EBSCOhost and Trip Medical Database were used.

**Results:**

Combined programs are predominate used for improving mood in elderly and the combinations used more frequently was short-term, light to moderate sub maximal aerobic exercise combined with dynamic resistance exercise following by Short-term, light to moderate sub maximal aerobic exercise combined with static exercise. Other psychosocial factors that commonly included in the combined exercise programs are group interaction, mindfulness and music.

**Conclusions:**

Combined exercise programs are as effective as simple programs in alleviated the symptoms of depression in elderly. Their advantages over simple programs are that they can promote other health benefits; they are less time consuming and more pleasurable to participants. Therefore, they are good exercise choice for elderly.

